# Venous invasion as a risk factor for recurrence after gastrectomy followed by chemotherapy for stage III gastric cancer

**DOI:** 10.1186/s12885-018-4052-z

**Published:** 2018-01-30

**Authors:** Keiji Nishibeppu, Shuhei Komatsu, Daisuke Ichikawa, Taisuke Imamura, Toshiyuki Kosuga, Kazuma Okamoto, Hirotaka Konishi, Atsushi Shiozaki, Hitoshi Fujiwara, Eigo Otsuji

**Affiliations:** Division of Digestive Surgery, Department of Surgery, Kyoto Prefectural University of Medicine, 465 Kajii-cho, Kawaramachihirokoji, Kamigyo-ku, Kyoto, 602-8566 Japan

**Keywords:** Gastric cancer, Venous invasion, Adjuvant chemotherapy, Chemoresistance, Hematogenous recurrence, Stage III

## Abstract

**Background:**

Although adjuvant chemotherapy with S-1 after curative gastrectomy has been performed as a standard treatment for Stage II and III gastric cancer (GC) in Japan, patients with Stage III GC still have a high incidence of recurrence and a poor prognostic outcome. The aim of this study was to investigate risk factors for recurrence in patients with Stage III GC despite of curative gastrectomy followed by adjuvant chemotherapy, suggesting an indicator for more intensive management.

**Methods:**

A total of 97 patients with pathological Stage III GC underwent adjuvant chemotherapy after curative gastrectomy between 2001 and 2014, were enrolled in this study. We retrospectively analyzed their hospital records from our hospital.

**Results:**

The 5-year relapse-free survival (RFS) rates of patients with pStage III GC were 42.0%. Univariate and multivariate analyses for RFS revealed that venous invasion (v+) was an independent factor predicting a shorter RFS (v + vs. v-, 36.5% vs. 47.4%, *P* = 0.034, HR 1.82, 95% CI: 1.01–3.37). Venous invasion also predicted a shorter overall survival (OS) (v + vs. v-, 33.7% vs. 50.4%, *P* = 0.027). Regarding the patterns of recurrence, hematogenous recurrence was significantly occurred in patients with v + GC than those without (*P* = 0.022).

**Conclusions:**

Stage III GC with venous invasion is a high-risk subgroup for hematogenous recurrence after curative surgery followed by adjuvant chemotherapy. More intensive and effective adjuvant chemo and/or molecular targeted therapy for Stage III GC patients with venous invasion should be considered to improve their outcomes.

**Electronic supplementary material:**

The online version of this article (10.1186/s12885-018-4052-z) contains supplementary material, which is available to authorized users.

## Background

Gastric cancer (GC) is a major cause of death worldwide [1]. Treatments for GC have certainly improved with recent advances in surgical procedures and adjuvant chemotherapy [2–5]. However, treatment of the primary tumor and regional lymph nodes is recognized as the only chance for macroscopic tumor clearance and cure for GC; the effects of such surgical resection is restricted to locally controlling the primary tumor [2, 3], and cannot prevent recurrence due to micro-metastasis, which could be residual and/or occur at the time of surgery [6, 7]. Therefore, perioperative systemic chemotherapy has been recommended to achieve microscopic tumor clearance.

Cunningham et al. [8] demonstrated that perioperative chemotherapy with a regimen of epirubicin, cisplatin, and fluorouracil improves overall survival (OS) and relapse-free survival (RFS) among patients with resectable adenocarcinoma of the stomach as compared with surgery alone. Intergroup 0116 demonstrated a notable benefit in OS and RFS by performing adjuvant chemo-radiotherapy following curative GC resection in patients with ≥ T3 and/or node-positive GC [9, 10]. In Japan, adjuvant chemotherapy following curative gastrectomy for stage II or III GC has been recommended as a standard treatment, based on the result of the ACTS-GC (Adjuvant Chemotherapy Trial of TS-1 for Gastric Cancer) [11, 12]. The ACTS-GC demonstrated the overall survival benefit (HR 0.67, 95% CI: 0.54–0.83) of adjuvant chemotherapy with S-1 in patients with stage II or III GC. With the proven survival benefit of perioperative adjuvant chemotherapy, it is now globally used [13].

According to the results of the ACTS-GC trial, the five-year OS rate of patients with stage II GC was 84.2%, whereas the five-year OS rate in stage IIIA and IIIB patients was only 57.3% and 44.1%, respectively [12]. Therefore, the efficacy of S-1 may be limited to stage II GC, with a need to improve the prognostic markers in stage III GC patients after combined curative gastrectomy and adjuvant S-1 chemotherapy in Japan. Several studies have recently been conducted to investigate the safety and efficacy of a more intensive combination chemotherapy as adjuvant therapy [14–16]. To validate these more intensive adjuvant chemo- and/or molecular-targeted therapies, stage III GC patients, with a high-risk factor for recurrence following adjuvant chemotherapy, need to be identified. In this study, to promote more intensive adjuvant treatment, we aimed to investigate surrogate pathologic factors for recurrence in stage III GC after curative gastrectomy followed by adjuvant chemotherapy.

## Methods

### Patients and surgical procedures

The study was approved by the Kyoto Prefectural University of Medicine and have therefore been performed in accordance with the ethical standards laid down in an appropriate version of the Declaration of Helsinki. Written informed consent was obtained from all patients for research purposes.

A total of 127 patients underwent curative gastrectomies, followed by adjuvant chemotherapy for pathological stage III GC at our institute between January 2001 and December 2014. All of the enrolled patients had undergone D2 or D2+ lymphadenectomies. In the D2 dissection, the perigastric lymph nodes and all second-tier lymph nodes were completely retrieved. Depending on the location of the tumor, the lymphadenectomy was done along the distal side of the splenic artery (No.11d) and at the splenic hilum (No.10), together with a splenectomy, or splenectomy with a distal pancreatectomy [17]. Enrolled patients also underwent macroscopic and pathologically curative resection (R0), and had negative results for peritoneal washing cytology.

Of these 127 patients, 30 patients were excluded from the study because of neoadjuvant chemotherapy (*n* = 28) and insufficient follow-up (n = 2). Consequently, a total of 97 patients were enrolled in this study. Resected specimens were examined by pathologists, and evaluated based on classifications of the 14th JCGC and 7th AJCC-UICC staging systems. All dissected lymph nodes were fixed in buffered formalin and embedded in paraffin and subjected to pathological examination. Pathologists in our institution examined embedded lymph nodes by sectioning slices in the plane of the largest node dimension to confirm the presence of metastasis. The clinicopathological findings of these patients were determined retrospectively on the basis of their hospital records.

### Follow-up after curative gastrectomy followed by adjuvant chemotherapy

Post-operative follow-ups were performed every three months after surgery in the outpatient clinic. Blood chemistry was measured every three months. Endoscopic examinations were performed annually, and computed tomography (CT) examinations were performed every three-to-six months for five years after surgery.

### Statistical analysis

The Chi-square test and Fisher’s exact probability test were used to analyze categorical variables to compare the clinicopathological characteristics between the two groups. For the analysis of survival, Kaplan-Meier survival curves were constructed for groups based on univariate predictors, and differences between the groups were tested with a generalized Wilcoxon test. The Cox proportional hazards model was used for further evaluations of multivariate survival analysis. A *p*-value < 0.05 was considered significant. Statistical analyses were conducted using JMP 10 (SAS Institute Inc., Cary, NC).

## Results

### Clinicopathological characteristics of stage III GC patients after curative gastrectomy followed by adjuvant chemotherapy

Table 1 shows the characteristics and treatment of patients with stage III GC in this study. The median age was 65 years old. Of these, 61 patients (63%) were male and 36 patients (37%) were female. Total gastrectomy was performed in 53 patients (55%) and distal gastrectomy in 44 patients (45%) as curative resection according to the location of the tumor to secure a sufficient resection margin. Of 97 stage III GC patients, 41 patients (42%) received S-1 alone, 12 patients (12%) received tegafur-uracil, 12 patients (12%) received methotrexate plus 5-fluorouracil, 14 patients (14%) received S-1 plus cisplatin, 5 patients (5%) received 5-fluorouracil alone, 7 patients (7%) received S-1 plus paclitaxel, 5 patients (5%) received 5-fluorouracil plus cisplatin and 1 patient (1%) received paclitaxel alone as adjuvant chemotherapy. None of the patients received adjuvant radiotherapy or chemo-radiotherapy.

**Table 1:** Clinical characteristics and treatment of stage III GC patients

Variables	Number	Percent
Total	97	
Gender		
Male	61	63%
Female	36	37%
Age		
≥65	51	53%
<65	46	47%
Tumor location		
U	19	20%
M	28	29%
L	32	33%
Whole	18	19%
Tumor major axis(mm)		
≥90	33	34%
<90	64	66%
T-stage		
T2 / T3	27	28%
T4	70	72%
N-stage		
N1/ N2	47	48%
N3	50	52%
Histopathological type		
Differentiated	31	32%
Undifferentiated	66	68%
Venous invasion		
Present	49	51%
Absent	48	49%
Lymphatic invasion		
Present	87	90%
Absent	10	10%
Surgical procedure		
Total gastrectomy	53	55%
Distal gastrectomy	44	45%

### Clinical outcomes and prognostic factors for relapse-free survival of stage III GC patients after curative gastrectomy followed by adjuvant chemotherapy

**Fig. 1:**
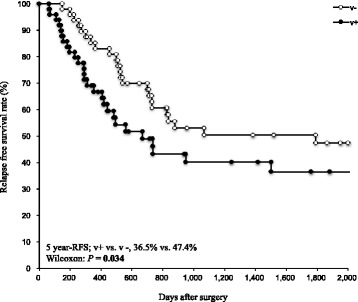
Relapse-free survival analysis of each group divided according to venous invasion. The patients with venous invasion exhibited a significantly poorer relapse-free survival (RFS) than those without (5-year RFS, 36.5% vs. 47.4%, *P* = 0.034)

**Fig. 2:**
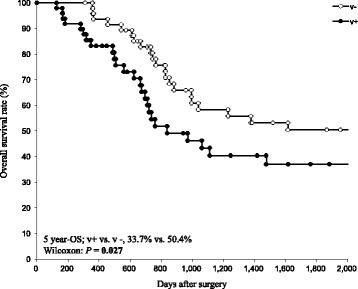
5-year overall survival analysis of each group divided according to venous invasion. The cumulative 5-year overall survival (OS) rate of the total 97 patients with Stage III gastric cancer was 42.6%. The patients with venous invasion exhibited a significantly poorer OS than patients with gastric cancer of other histological types (5-year OS, 33.7% vs. 50.4%, *P* = 0.027)

The average observation period was 43.9 months. The cumulative five-year RFS and OS rates in the 97 patients with stage III GC were 42.0%, and 42.6%, respectively. Univariate analysis revealed venous invasion (*P* = 0.034) as a prognostic factor for RFS after curative gastrectomy followed by adjuvant chemotherapy. Multivariate analysis using the Cox proportional hazard model demonstrated that venous invasion was the only independent factor predicting a shorter RFS in stage III GC (*P* = 0.048, HR 1.82, 95% CI: 1.01–3.37) (Table 2). Fig. 1 shows the RFS curves according to GC patients with or without venous invasion. A significant difference was observed between patients with and without venous invasion (Figs. 1, 5-year RFS rates, venous invasion + vs. venous invasion -; 36.5% vs. 47.4%, *P* = 0.034). Furthermore, GC patients with venous invasion exhibited a shorter OS than those with without GC (Figs. 2, 5-year OS rates, venous invasion + vs. venous invasion -; 33.7% vs. 50.4%, *P* = 0.027). Additional file 1**:** Figure S1 shows the RFS and OS curves for GC patients after curative gastrectomy followed by S-1 treatment alone with or without venous invasion. No significant difference was observed between patients with or without venous invasion (Additionral file 1: Figure S1a, 5-year RFS rates, venous invasion + vs. venous invasion -, 41.8% vs. 41.5%, *P* = 0.089; Additional file 2: Figure S1b, 5-year OS rates, venous invasion + vs. venous invasion -, 36.4% vs. 58.0%, *P* = 0.163). However, the prognoses of GC patients after curative gastrectomy followed by S-1 treatment alone with venous invasion tended to be poorer than those of patients without venous invasion.

**Table 2:** Univariate and multivariate analysis for relapse-free survival (RFS) in stage III GC patients after curative gastrectomy followed by adjuvant chemotherapy

				Univariate^a^	Multivariate^b^		
		N	5-year RFS^c^	*P*-value	HR^d^	95% CI^e^	*P*-value
Sex	Male	61	46.1%	0.577	1.20	0.63–2.31	0.581
	Female	36	36.0%				
Age	<65	46	42.2%	0.824	1.39	0.76–2.53	0.285
	≥65	51	41.9%				
Tumor major axis(mm)	≥90	33	30.8%	0.092	1.46	0.71–3.21	0.109
	<90	64	48.8%				
T-stage	T4	70	38.9%	0.326	1.46	0.79–3.63	0.311
	T2/ T3	27	50.1%				
N-stage	N3	50	33.4%	0.339	1.51	0.82–2.84	0.187
	N1/ N2	47	50.9%				
Venous invasion	Present	48	36.5%	**0.034**	1.82	1.01–3.37	**0.048**
	Absent	49	47.4%				
Lymphatic invasion	Present	87	39.8%	0.168	1.51	0.56–5.28	0.445
	Absent	10	40.0%				

^a^Kaplan-Meier method: significance was determined by Wilcoxon test

^b^Multivariate survival analysis was performed using Cox’s proportional hazard model

^c^*RFS* Relative free survival

^d^*HR* Hazard ratio

^e^*CI* Confidence interval

Significant values are in bold

### Comparison of recurrence patterns according to stage III GC patients with or without venous invasion

Table 3 shows postoperative adjuvant treatments according to the status of venous invasion in stage III GC patients. There was no significant difference between patients with or without venous invasion in the regimens of adjuvant chemotherapy. Regarding the patterns of recurrence, the incidence of hematogenous recurrence was significantly higher in patients with venous invasion than in those without (venous invasion + vs. venous invasion -: 10% vs. 2%, *P* = 0.022; Table 4).

**Table 3:** The regimens of adjuvant chemotherapy

			Venous invasion		
		N	Present	Absent	*P*-value
Regimen					
	S-1	41	23(24%)	18(19%)	0.346
	S-1 plus cisplatin	14	10(10%)	4(4%)	0.086
	5-FU plus methotrexate	12	4(4%)	8(8%)	0.200
	UFT	12	5(5%)	7(7%)	0.511
	S-1 plus paclitaxel	7	2(2%)	5(5%)	0.221
	5-FU plus cisplatin	5	3(3%)	2(2%)	0.662
	5-FU	5	1(1%)	4(4%)	0.148
	Paclitaxel	1	1(1%)	0(0%)	0.241

**Table 4:** Comparison of recurrence patterns according to stage III GC patients with or without venous invasion

			Venous invasion		Univariate^a^
		N	Present	Absent	*P*-value
Total		97	49 (51%)	48 (49%)	0.615
Hematogenous recurrence		12	10 (10%)	2 (2%)	**0.022**
	Liver	8	7 (7%)	1 (1%)	**0.021**
	Lung	2	2 (2%)	0 (0%)	0.096
	Bone	2	1 (1%)	1 (1%)	0.988
Lymphatic recurrence		12	7 (7%)	5 (5%)	0.562
Peritoneal recurrence		34	18 (19%)	16 (16%)	0.726
Local recurrence		4	1 (1%)	3 (3%)	0.287

^a^Kaplan-Meier method: significance was determined by the Wilcoxon test

Significant values are in bold

## Discussion

This study demonstrated that stage III GC patients with venous invasion have a markedly poor prognosis despite curative gastrectomy followed by adjuvant chemotherapy. Furthermore, this study clearly identified stage III GC patients with venous invasion as a high-risk subgroup for hematogenous recurrence. These results strongly indicate that GC with venous invasion could be specifically targeted in an effort to improve the prognosis of patients with stage III GC, suggesting an indication for more intensive adjuvant chemo- and/or molecular targeted therapy.

Based on the results of the ACTS-GC trial for stage II or III GC, conventional adjuvant chemotherapy with S-1 has the potential to reduce the incidence of peritoneal and lymphatic recurrences. However, this trial also indicated the limitations of the inhibitory effects of S-1 on hematogenous recurrence [11], particularly in stage III GC. To further improve the prognostic outcomes, the prevention of hematogenous recurrence after surgery should be a pivotal treatment target in advanced GC patients. Hematogenous metastasis results when cancer cells derived from the primary lesion enter blood vessels and are transported to distant organs where they can proliferate and form secondary tumors. This leads to hematogenous recurrence. Several previous reports demonstrated that vascular invasion in resected specimens was a risk factor for hematogenous recurrence and a poor prognostic factor in GC [18, 19]. These results strongly support our results that GC with venous invasion is a high-risk subgroup for hematogenous recurrence after curative gastrectomy followed by adjuvant chemotherapy. Thus, we suggest that venous invasion is the surrogate biomarker for an indication of more intensive chemo- and/or molecular-targeted therapy in order to prevent hematogenous recurrence.

Recently, several promising studies have been conducted to investigate the efficacy of combination chemotherapy as a more intensive adjuvant therapy for stage III GC [14–16, 20, 21]. Of these, the results of the CLASSIC trial indicated that adjuvant treatment with capecitabine plus oxaliplatin may have the potential to reduce the incidence of hematogenous recurrence [22]. Currently, this regimen is already included in standard adjuvant chemotherapy for stage II and III GC in Japan. Furthermore, the efficacy of molecular targeted drugs against tumor angiogenesis, which is essential for hematogenous metastasis, has been demonstrated [23–25]. Trastuzumab in combination with chemotherapy for patients with HER2-positive advanced gastric or gastro-esophageal junction cancer was proven to be more effective for measurable tumors such as hematogenous recurrence [23]. Ohtsu et al. [24] demonstrated that adding bevacizumab, a monoclonal antibody targeting VEGF-A, to fluoropyrimidine-cisplatin improved progression-free survival and overall response rate in the first-line treatment of advanced GC. The Rainbow trial indicated that adding ramucirumab, a monoclonal antibody VEGFR-2 antagonist, to paclitaxel improved OS in 665 GC patients (median 9.6 months vs 7.4 months stratified) (*P =* 0.017, HR 0.81, 95% CI: 0.68–0.96) [25]. These treatment strategies for advanced GC may become treatment candidates as more intensive adjuvant therapies for stage III GC with venous invasion.

This study is limited because the results were obtained from a retrospective evaluation with a small number of patients and inconsistent treatments at a single institute. A large-scale or multicenter prospective cohort study is warranted to validate the significance of the strategy.

## Conclusion

We demonstrated that stage III GC with venous invasion presented a worse prognosis and higher rate of hematogenous recurrence despite adjuvant chemotherapy. Stage III GC with venous invasion could be specifically targeted in an effort to improve the prognosis with stage III GC, suggesting an indication for additional adjuvant chemotherapy.

## Additional files

Additional file 1: Figure S1a. Relapse-free survival curves according to the status of venous invasion in GC patients after curative gastrectomy followed by S-1 treatment alone. No significant difference was observed between patients (+)venous invasion or (−) venous invasion (5-year RFS: 41.8% vs. 41.5%, P = 0.089) (PDF 49 kb)

Additional file 2: Figure S1b. Overall survival curves according to venous invasion status of GC patients after curative gastrectomy followed by S-1 treatment alone. No significant difference was observed between patients (+)venous invasion or (−) venous invasion (5-year OS: 36.4% vs. 58.0%, *P* = 0.163) (PDF 49 kb)

## Abbreviations

ACTS-GC: Adjuvant Chemotherapy Trial of TS-1 for Gastric Cancer
CT: Computed tomography
GC: Gastric cancer
RFS: Relapse-free survival
UFT: Uracil tegafur
v+: Venous invasion
