# Supplementary figures and images for: Venous invasion as a risk factor for recurrence after gastrectomy followed by chemotherapy for stage III gastric cancer
Source: BMC Cancer. 2018 Jan 30;18:108. doi: 10.1186/s12885-018-4052-z (PMC5791734; doi:10.1186/s12885-018-4052-z)

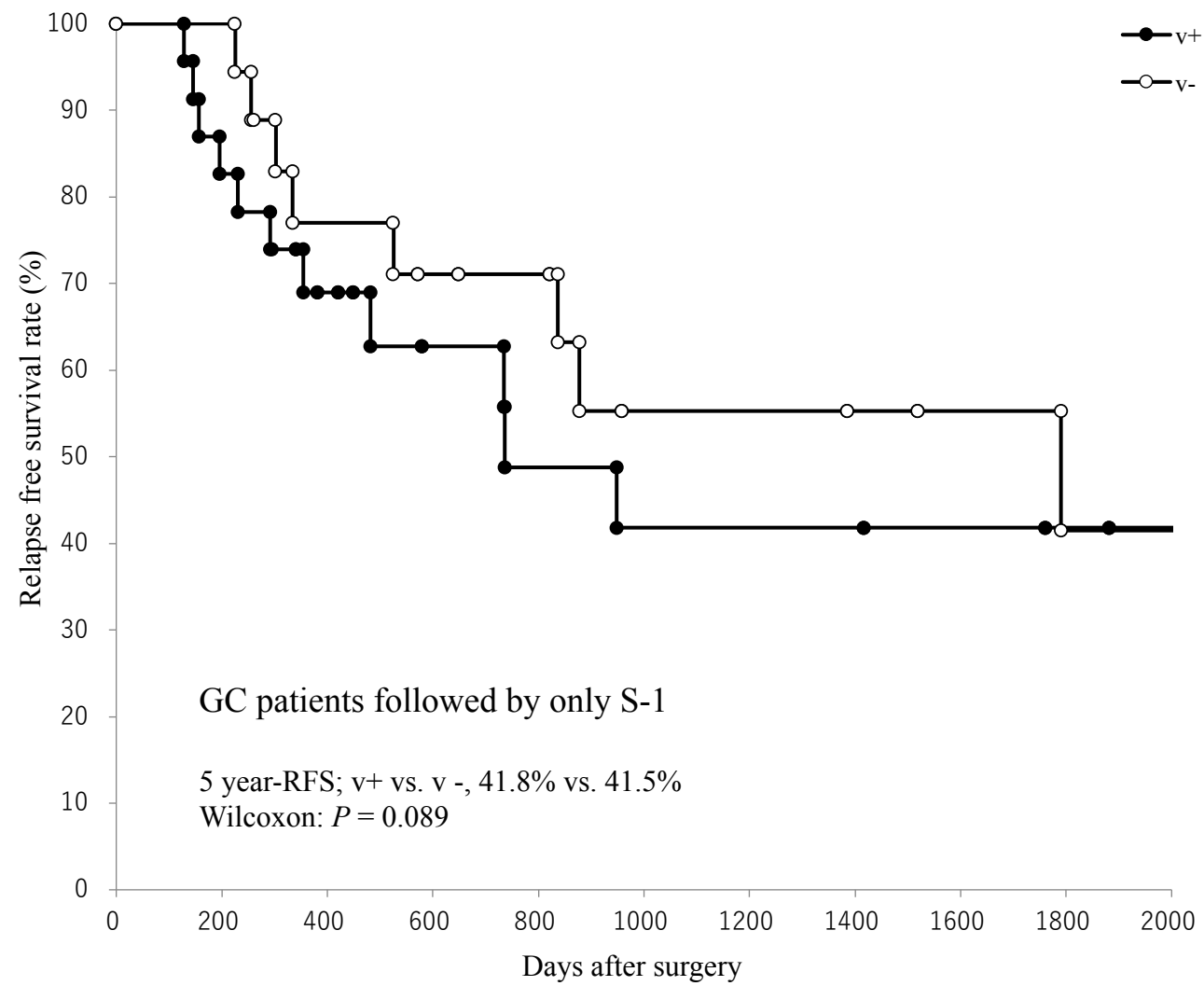

Supplement Fig. 1a

Supplement: Supplementary file 1 — Relapse-free survival curves according to the status of venous invasion in GC patients after curative gastrectomy followed by S-1 treatment alone. No significant difference was observed between patients (+)venous invasion or (−) venous invasion (5-year RFS: 41.8% vs. 41.5%, P = 0.089) (PDF 49 kb) [file 12885_2018_4052_MOESM1_ESM.pdf]

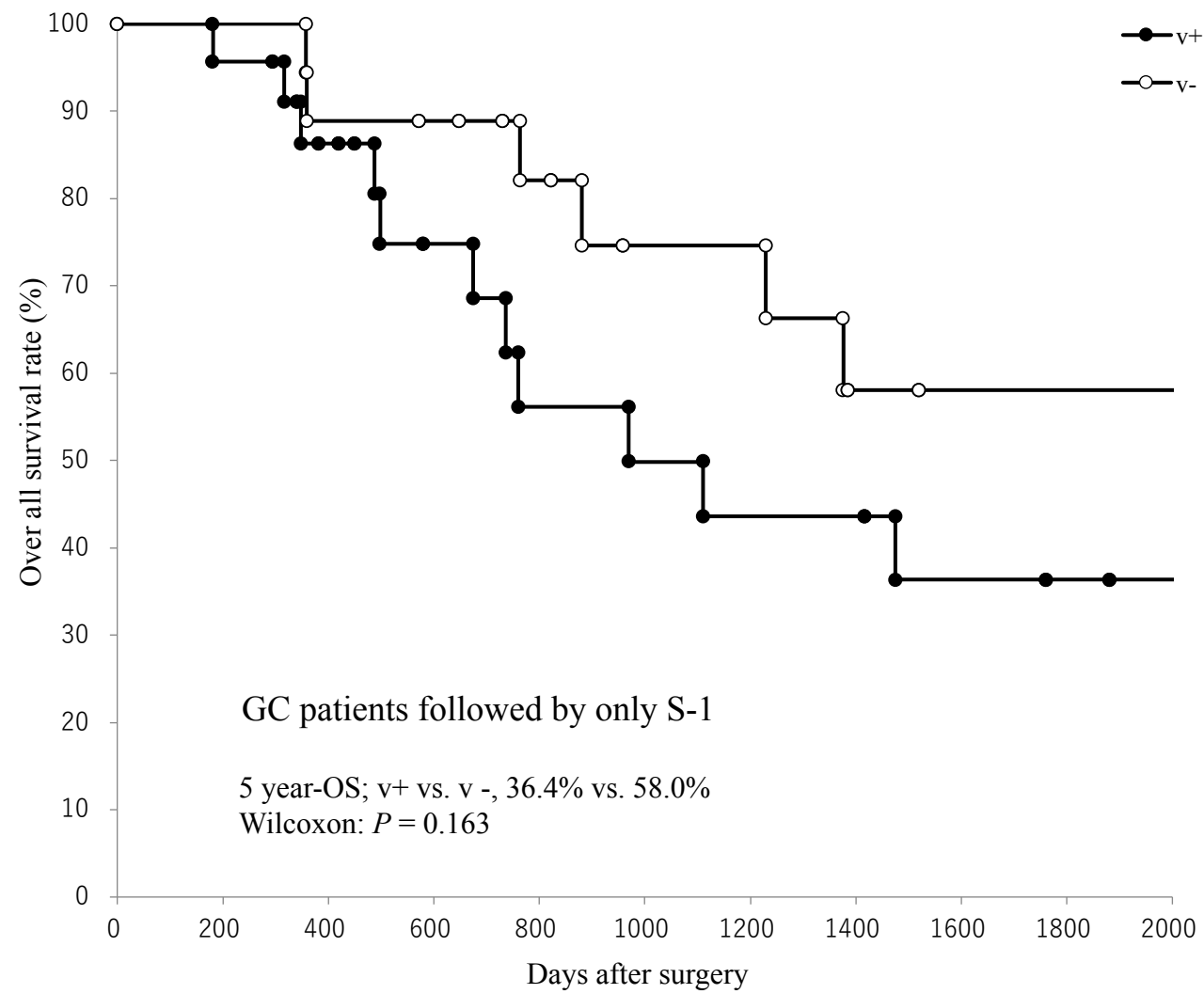

Supplement Fig. 1b

Supplement: Supplementary file 2 — Overall survival curves according to venous invasion status of GC patients after curative gastrectomy followed by S-1 treatment alone. No significant difference was observed between patients (+)venous invasion or (−) venous invasion (5-year OS: 36.4% vs. 58.0%, P = 0.163) (PDF 49 kb) [file 12885_2018_4052_MOESM2_ESM.pdf]
